# 6-Benzyl-3-(1,4-dioxaspiro­[4.5]decan-2-yl)-8,8-dimethyl-1-oxa-2,6-diaza­spiro­[4.4]non-2-ene-7,9-dione

**DOI:** 10.1107/S1600536809044675

**Published:** 2009-10-31

**Authors:** Yaser Bathich, Zurina Shaameri, Ahmad Sazali Hamzah, Ching Kheng Quah, Hoong-Kun Fun

**Affiliations:** a Institute of Science, Universiti Teknologi MARA, 40450 Shah Alam, Selangor, Malaysia; b X-ray Crystallography Unit, School of Physics, Universiti Sains Malaysia, 11800 USM, Penang, Malaysia

## Abstract

In the title compound, C_23_H_28_N_2_O_5_, the 4,5-dihydro­isoxazole ring adopts a slight envelope conformation and the dioxolane ring is in a twisted conformation. The mol­ecular structure, in the vicinity of the benzyl group, may be influenced by an intra­molecular C—H⋯O hydrogen bond which generates an *S*(7) ring motif. In the crystal structure, mol­ecules are linked *via* weak inter­molecular C—H⋯O hydrogen bonds, forming extended chains along the *b* axis. Further stabilization is provided by weak C—H⋯π inter­actions.

## Related literature

For general background to and applications of pyrrolidinone derivatives, see: Iida *et al.* (1986 ▶); Matkhalikova *et al.* (1969 ▶); Reddy & Rao (2006 ▶); Reiner (2007 ▶); Royles (1996 ▶); Sauleau & Bourguet-Kondracki (2005 ▶). For a related structure, see: Bathich *et al.* (2009 ▶). For a description of hydrogen-bond motifs, see: Bernstein *et al.* (1995 ▶). For the definition of ring conformations, see: Cremer & Pople (1975 ▶). For the stability of the temperature controller used for the data collection, see: Cosier & Glazer (1986 ▶). 
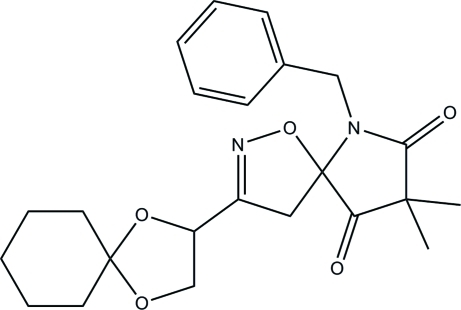


## Experimental

### 

#### Crystal data

C_23_H_28_N_2_O_5_
*M* *_r_* = 412.47
Orthorhombic, 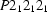

*a* = 9.8666 (2) Å
*b* = 11.1565 (3) Å
*c* = 18.4884 (4) Å
*V* = 2035.14 (8) Å^3^
*Z* = 4
Mo *K*α radiation
μ = 0.10 mm^−1^
*T* = 100 K
0.49 × 0.23 × 0.16 mm

#### Data collection

Bruker SMART APEXII CCD area-detector diffractometer
Absorption correction: multi-scan (**SADABS**; Bruker, 2005 ▶) *T*_min_ = 0.931, *T*_max_ = 0.985
26273 measured reflections
4621 independent reflections
4013 reflections with *I* > 2σ(*I*)
*R*_int_ = 0.034

#### Refinement

*R*[*F* ^2^ > 2σ(*F* ^2^)] = 0.040
*wR*(*F* ^2^) = 0.099
*S* = 1.06
4621 reflections
273 parameters
H-atom parameters constrained
Δρ_max_ = 0.27 e Å^−3^
Δρ_min_ = −0.26 e Å^−3^

### 

Data collection: *APEX2* (Bruker, 2005 ▶); cell refinement: *SAINT* (Bruker, 2005 ▶); data reduction: *SAINT*; program(s) used to solve structure: *SHELXTL* (Sheldrick, 2008 ▶); program(s) used to refine structure: *SHELXTL*; molecular graphics: *SHELXTL*; software used to prepare material for publication: *SHELXTL* and *PLATON* (Spek, 2009 ▶).

## Supplementary Material

Crystal structure: contains datablocks global, I. DOI: 10.1107/S1600536809044675/lh2930sup1.cif

Structure factors: contains datablocks I. DOI: 10.1107/S1600536809044675/lh2930Isup2.hkl

Additional supplementary materials:  crystallographic information; 3D view; checkCIF report

## Figures and Tables

**Table 1:** Hydrogen-bond geometry (Å, °)

*D*—H⋯*A*	*D*—H	H⋯*A*	*D*⋯*A*	*D*—H⋯*A*
C1—H1*A*⋯O1	0.93	2.48	3.1440 (18)	129
C14—H14*A*⋯O1^i^	0.98	2.50	3.4692 (17)	171
C15—H15*A*⋯*Cg*^ii^	0.97	2.89	3.6484 (15)	136

Symmetry codes: (i) 
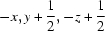
; (ii) 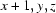. *Cg*1 is the centroid of C1–C6 ring.

**Fig. 1.**
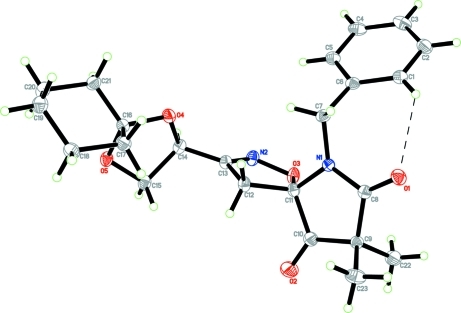
The molecular structure of the title compound, showing 50% probability displacement ellipsoids for non-H atoms and the atom-numbering scheme. A weak intramolecular interaction is shown as dashed line.

**Fig. 2.**
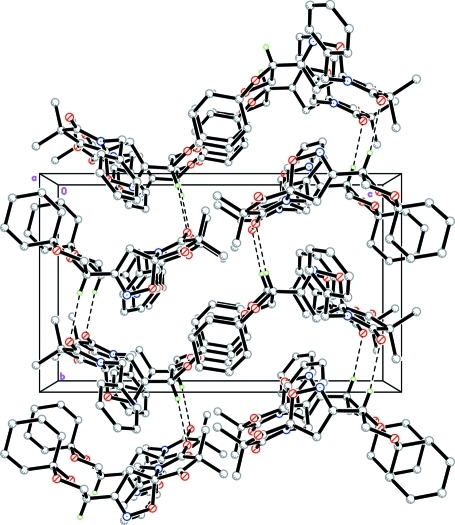
Part of the crystal structure of the title compound viewed along the a axis. H atoms not involved in intermolecular interactions (dashed lines) have been omitted for clarity.

## supplementary crystallographic information

### Comment

Natural products containing pyrrolidinone carbon skeletons continue to attract the interest of chemists and biologists due to their challenging structures and remarkable biological properties (Iida *et al.*, 1986; Matkhalikova *et al.*, 1969; Reddy & Rao, 2006; Reiner, 2007; Royles, 1996). Amongst these, polychlorinated pyrrolidinone *i.e.* dysidamide analogues extracted from the marine sponge, Lamellodysidea herbacea, display remarkable biological activities (Sauleau & Bourguet-Kondracki, 2005). We have synthesized the title compound, which may act as an essential intermediate in the synthesis of dysidamide and its crystal structure is reported herein.

The molecular structure of the title compound is shown in Fig. 1. The 4,5-dihydroisoxazole ring (O3/N2/C11–C13) adopts an envelope conformation with atom C11 displaced from the mean plane by 0.045 (1) Å; the puckering parameters (Cremer & Pople, 1975) are Q = 0.0730 (13) Å and Θ = 330.2 (11)°  whereas the conformation of dioxolane ring (O4/O5/C14–C16) is twisted, as reflected by the puckering parameters, Q = 0.3405 (13) Å and Θ = 82.9 (2)°, with torsion angle C16–O5–C15–C14 being -36.76 (12) °. The molecular structure is stabilized by an intramolecular C1—H1A···O1 hydrogen bond which generates an *S*(7) ring motif (Bernstein *et al.*, 1995). Bond lengths and angles are within normal ranges, and comparable to a closely related structure (Bathich *et al.*, 2009).

In the solid state (Fig. 2), the molecules are linked *via* intermolecular C14—H14A···O1^i^ hydrogen bonds to form one-dimensional chains along the *b*-axis and are further consolidated by C–H···π (Table 1) interactions.

### Experimental

935 mg (4.25 mmol) of the hydroximoyl chloride at 273 K was dissolved in 100 ml of diethyl ether and 650 mg (2.84 mmol) of *N*-protected-5-methylene-pyrrolidine-2,4-dione was added. To this mixture 9.35 ml (0.5 M, 4.675 mmol) of triethylamine solution in ether was added dropwise at a rate of 8 to 10 drops/min over 4h and stirred overnight. The mixture was then quenched by addition of 100ml HCl (2 N) and partitioned against ether (4 x 60 ml). The combined organic phases were washed with NaHCO_3_ (100 ml) and water (2 x 100 ml), then dried with MgSO_4_, and concentrated in vacuo (15 mbar) to give a yellow oil. Crystallization from diethyl ether gave the analytically and spectroscopically pure spiroisoxazoline (880 mg, 75 %) as colourless crystals. *M.p.* 403–404 K.

### Refinement

All H atoms were placed in the calculated positions, with C–H = 0.93–0.98 Å, and refined using a riding model, with *U*_iso_(H) = 1.2 or 1.5 *U*_eq_(C). A rotating-group model was applied for the methyl groups. In the absence of significant anomalous dispersion, 3651 Friedel pairs were merged for the final refinement.

### Crystal data

**Table:**

C_23_H_28_N_2_O_5_	*F*(000) = 880
*M**_r_* = 412.47	*D*_x_ = 1.346 Mg m^−^^3^
Orthorhombic, *P*2_1_2_1_2_1_	Mo *K*α radiation, λ = 0.71073 Å
Hall symbol: P 2ac 2ab	Cell parameters from 9915 reflections
*a* = 9.8666 (2) Å	θ = 2.1–33.4°
*b* = 11.1565 (3) Å	µ = 0.10 mm^−^^1^
*c* = 18.4884 (4) Å	*T* = 100 K
*V* = 2035.14 (8) Å^3^	Block, colourless
*Z* = 4	0.49 × 0.23 × 0.16 mm

### Data collection

**Table:**

Bruker SMART APEXII CCD area-detector diffractometer	4621 independent reflections
Radiation source: fine-focus sealed tube	4013 reflections with *I* > 2σ(*I*)
graphite	*R*_int_ = 0.034
φ and ω scans	θ_max_ = 34.0°, θ_min_ = 2.1°
Absorption correction: multi-scan (*SADABS*; Bruker, 2005)	*h* = −12→15
*T*_min_ = 0.931, *T*_max_ = 0.985	*k* = −17→16
26273 measured reflections	*l* = −29→27

### Refinement

**Table:**

Refinement on *F*^2^	Primary atom site location: structure-invariant direct methods
Least-squares matrix: full	Secondary atom site location: difference Fourier map
*R*[*F*^2^ > 2σ(*F*^2^)] = 0.040	Hydrogen site location: inferred from neighbouring sites
*wR*(*F*^2^) = 0.099	H-atom parameters constrained
*S* = 1.06	*w* = 1/[σ^2^(*F*_o_^2^) + (0.053*P*)^2^ + 0.1506*P*] where *P* = (*F*_o_^2^ + 2*F*_c_^2^)/3
4621 reflections	(Δ/σ)_max_ < 0.001
273 parameters	Δρ_max_ = 0.27 e Å^−^^3^
0 restraints	Δρ_min_ = −0.26 e Å^−^^3^

### Special details

**Table:**

Experimental. The crystal was placed in the cold stream of an Oxford Cyrosystems Cobra open-flow nitrogen cryostat (Cosier & Glazer, 1986) operating at 100.0 (1) K.
Geometry. All esds (except the esd in the dihedral angle between two l.s. planes) are estimated using the full covariance matrix. The cell esds are taken into account individually in the estimation of esds in distances, angles and torsion angles; correlations between esds in cell parameters are only used when they are defined by crystal symmetry. An approximate (isotropic) treatment of cell esds is used for estimating esds involving l.s. planes.
Refinement. Refinement of F^2^ against ALL reflections. The weighted R-factor wR and goodness of fit S are based on F^2^, conventional R-factors R are based on F, with F set to zero for negative F^2^. The threshold expression of F^2^ > 2sigma(F^2^) is used only for calculating R-factors(gt) etc. and is not relevant to the choice of reflections for refinement. R-factors based on F^2^ are statistically about twice as large as those based on F, and R- factors based on ALL data will be even larger.

### Fractional atomic coordinates and isotropic or equivalent isotropic displacement parameters (Å^2^)

**Table:**

	*x*	*y*	*z*	*U*_iso_*/*U*_eq_	
O1	−0.23832 (10)	0.26330 (9)	0.40562 (6)	0.0196 (2)	
O2	0.22502 (11)	0.36138 (13)	0.40410 (7)	0.0325 (3)	
O3	0.05398 (10)	0.52460 (9)	0.31291 (5)	0.0187 (2)	
O4	0.14033 (10)	0.38771 (9)	0.09244 (5)	0.0196 (2)	
O5	0.36514 (10)	0.35805 (9)	0.06709 (5)	0.01736 (19)	
N1	−0.09667 (11)	0.35996 (10)	0.32684 (6)	0.0143 (2)	
N2	0.11326 (13)	0.55491 (11)	0.24486 (6)	0.0186 (2)	
C1	−0.41547 (14)	0.44109 (13)	0.31359 (8)	0.0190 (3)	
H1A	−0.4219	0.3748	0.3439	0.023*	
C2	−0.51858 (15)	0.52627 (15)	0.31315 (8)	0.0227 (3)	
H2A	−0.5929	0.5173	0.3436	0.027*	
C3	−0.51064 (16)	0.62426 (15)	0.26739 (9)	0.0250 (3)	
H3A	−0.5796	0.6811	0.2672	0.030*	
C4	−0.39985 (15)	0.63778 (13)	0.22180 (8)	0.0224 (3)	
H4A	−0.3951	0.7031	0.1906	0.027*	
C5	−0.29601 (14)	0.55362 (13)	0.22283 (8)	0.0183 (2)	
H5A	−0.2215	0.5634	0.1926	0.022*	
C6	−0.30241 (13)	0.45449 (12)	0.26879 (7)	0.0156 (2)	
C7	−0.19254 (14)	0.36013 (12)	0.26595 (7)	0.0165 (2)	
H7A	−0.2354	0.2820	0.2636	0.020*	
H7B	−0.1416	0.3710	0.2216	0.020*	
C8	−0.12761 (13)	0.30580 (11)	0.39112 (7)	0.0146 (2)	
C9	−0.00312 (13)	0.30177 (12)	0.43963 (7)	0.0149 (2)	
C10	0.10534 (14)	0.35446 (13)	0.39145 (7)	0.0188 (3)	
C11	0.04302 (13)	0.39495 (12)	0.31910 (7)	0.0155 (2)	
C12	0.11909 (14)	0.34641 (12)	0.25325 (7)	0.0169 (2)	
H12A	0.2014	0.3050	0.2673	0.020*	
H12B	0.0627	0.2927	0.2250	0.020*	
C13	0.14980 (14)	0.45896 (12)	0.21270 (7)	0.0158 (2)	
C14	0.21716 (14)	0.46231 (12)	0.14013 (7)	0.0171 (2)	
H14A	0.2206	0.5446	0.1217	0.021*	
C15	0.35852 (14)	0.40534 (13)	0.13854 (7)	0.0185 (3)	
H15A	0.4287	0.4647	0.1466	0.022*	
H15B	0.3668	0.3424	0.1745	0.022*	
C16	0.23247 (14)	0.31499 (12)	0.05037 (7)	0.0158 (2)	
C17	0.21576 (14)	0.18392 (12)	0.07127 (7)	0.0174 (2)	
H17A	0.1211	0.1616	0.0670	0.021*	
H17B	0.2425	0.1733	0.1214	0.021*	
C18	0.30090 (15)	0.10213 (12)	0.02350 (7)	0.0193 (3)	
H18A	0.2804	0.0193	0.0350	0.023*	
H18B	0.3961	0.1156	0.0337	0.023*	
C19	0.27448 (16)	0.12392 (13)	−0.05699 (8)	0.0217 (3)	
H19A	0.3355	0.0748	−0.0855	0.026*	
H19B	0.1823	0.1007	−0.0687	0.026*	
C20	0.29553 (16)	0.25566 (13)	−0.07607 (8)	0.0210 (3)	
H20A	0.3899	0.2768	−0.0687	0.025*	
H20B	0.2741	0.2682	−0.1267	0.025*	
C21	0.20631 (15)	0.33598 (13)	−0.02966 (7)	0.0196 (3)	
H21A	0.2244	0.4192	−0.0413	0.023*	
H21B	0.1118	0.3198	−0.0403	0.023*	
C22	−0.02114 (16)	0.37625 (15)	0.50857 (8)	0.0241 (3)	
H22A	0.0582	0.3686	0.5383	0.036*	
H22B	−0.0988	0.3479	0.5347	0.036*	
H22C	−0.0341	0.4589	0.4959	0.036*	
C23	0.02932 (16)	0.17141 (13)	0.45856 (9)	0.0242 (3)	
H23A	0.1118	0.1682	0.4860	0.036*	
H23B	0.0399	0.1259	0.4149	0.036*	
H23C	−0.0434	0.1383	0.4867	0.036*	

### Atomic displacement parameters (Å^2^)

**Table:**

	*U*^11^	*U*^22^	*U*^33^	*U*^12^	*U*^13^	*U*^23^
O1	0.0155 (4)	0.0204 (5)	0.0228 (5)	−0.0023 (4)	0.0010 (4)	0.0025 (4)
O2	0.0156 (5)	0.0515 (8)	0.0306 (6)	−0.0057 (5)	−0.0042 (5)	0.0077 (6)
O3	0.0239 (5)	0.0135 (4)	0.0186 (4)	−0.0025 (4)	0.0067 (4)	−0.0024 (4)
O4	0.0170 (5)	0.0228 (5)	0.0190 (4)	0.0046 (4)	−0.0003 (4)	−0.0048 (4)
O5	0.0158 (4)	0.0199 (4)	0.0164 (4)	−0.0033 (4)	0.0021 (4)	−0.0030 (4)
N1	0.0132 (5)	0.0163 (5)	0.0134 (5)	−0.0007 (4)	0.0000 (4)	0.0004 (4)
N2	0.0215 (6)	0.0161 (5)	0.0182 (5)	−0.0012 (4)	0.0035 (4)	0.0004 (4)
C1	0.0174 (6)	0.0222 (6)	0.0175 (6)	−0.0005 (5)	−0.0017 (5)	−0.0007 (5)
C2	0.0168 (6)	0.0278 (7)	0.0235 (7)	0.0030 (5)	−0.0013 (5)	−0.0047 (6)
C3	0.0192 (7)	0.0224 (7)	0.0334 (8)	0.0042 (5)	−0.0067 (6)	−0.0037 (6)
C4	0.0228 (7)	0.0163 (6)	0.0280 (7)	−0.0013 (5)	−0.0091 (6)	0.0015 (5)
C5	0.0179 (6)	0.0178 (6)	0.0193 (6)	−0.0018 (5)	−0.0026 (5)	−0.0008 (5)
C6	0.0158 (6)	0.0160 (5)	0.0151 (5)	−0.0001 (4)	−0.0035 (5)	−0.0029 (4)
C7	0.0186 (6)	0.0165 (5)	0.0145 (5)	0.0026 (5)	−0.0032 (5)	−0.0030 (4)
C8	0.0160 (6)	0.0125 (5)	0.0154 (5)	0.0006 (4)	0.0002 (5)	−0.0007 (4)
C9	0.0148 (6)	0.0155 (6)	0.0144 (5)	0.0006 (4)	−0.0011 (5)	−0.0009 (4)
C10	0.0162 (6)	0.0209 (6)	0.0192 (6)	−0.0012 (5)	−0.0002 (5)	0.0009 (5)
C11	0.0145 (6)	0.0155 (5)	0.0165 (6)	−0.0012 (4)	0.0023 (5)	0.0004 (5)
C12	0.0193 (6)	0.0129 (5)	0.0186 (6)	0.0014 (4)	0.0051 (5)	0.0009 (4)
C13	0.0168 (6)	0.0138 (5)	0.0168 (6)	−0.0009 (4)	0.0016 (5)	0.0004 (4)
C14	0.0221 (6)	0.0142 (5)	0.0150 (5)	−0.0009 (5)	0.0023 (5)	−0.0002 (4)
C15	0.0185 (6)	0.0201 (6)	0.0168 (6)	−0.0032 (5)	0.0017 (5)	−0.0028 (5)
C16	0.0143 (6)	0.0169 (5)	0.0161 (6)	0.0012 (4)	0.0004 (5)	−0.0006 (4)
C17	0.0167 (6)	0.0177 (6)	0.0178 (6)	−0.0003 (5)	0.0014 (5)	0.0022 (5)
C18	0.0190 (6)	0.0160 (6)	0.0228 (6)	0.0014 (5)	−0.0004 (5)	0.0000 (5)
C19	0.0244 (7)	0.0205 (6)	0.0201 (6)	0.0018 (5)	−0.0003 (6)	−0.0036 (5)
C20	0.0252 (7)	0.0218 (6)	0.0162 (6)	0.0004 (5)	0.0023 (5)	−0.0007 (5)
C21	0.0236 (7)	0.0195 (6)	0.0155 (6)	0.0029 (5)	−0.0011 (5)	0.0006 (5)
C22	0.0239 (7)	0.0290 (7)	0.0193 (6)	0.0045 (6)	−0.0026 (6)	−0.0071 (6)
C23	0.0243 (7)	0.0155 (6)	0.0329 (8)	0.0021 (5)	−0.0076 (6)	0.0027 (6)

### Geometric parameters (Å, °)

**Table:**

O1—C8	1.2207 (16)	C11—C12	1.5293 (18)
O2—C10	1.2063 (18)	C12—C13	1.4935 (18)
O3—N2	1.4281 (15)	C12—H12A	0.9700
O3—C11	1.4550 (16)	C12—H12B	0.9700
O4—C14	1.4300 (17)	C13—C14	1.4978 (18)
O4—C16	1.4456 (16)	C14—C15	1.533 (2)
O5—C15	1.4239 (16)	C14—H14A	0.9800
O5—C16	1.4282 (17)	C15—H15A	0.9700
N1—C8	1.3676 (17)	C15—H15B	0.9700
N1—C11	1.4396 (17)	C16—C21	1.5200 (19)
N1—C7	1.4705 (16)	C16—C17	1.5214 (19)
N2—C13	1.2765 (17)	C17—C18	1.5227 (19)
C1—C2	1.392 (2)	C17—H17A	0.9700
C1—C6	1.3974 (19)	C17—H17B	0.9700
C1—H1A	0.9300	C18—C19	1.530 (2)
C2—C3	1.385 (2)	C18—H18A	0.9700
C2—H2A	0.9300	C18—H18B	0.9700
C3—C4	1.389 (2)	C19—C20	1.526 (2)
C3—H3A	0.9300	C19—H19A	0.9700
C4—C5	1.390 (2)	C19—H19B	0.9700
C4—H4A	0.9300	C20—C21	1.521 (2)
C5—C6	1.3961 (19)	C20—H20A	0.9700
C5—H5A	0.9300	C20—H20B	0.9700
C6—C7	1.5120 (19)	C21—H21A	0.9700
C7—H7A	0.9700	C21—H21B	0.9700
C7—H7B	0.9700	C22—H22A	0.9600
C8—C9	1.5216 (18)	C22—H22B	0.9600
C9—C10	1.5113 (19)	C22—H22C	0.9600
C9—C23	1.530 (2)	C23—H23A	0.9600
C9—C22	1.532 (2)	C23—H23B	0.9600
C10—C11	1.5399 (19)	C23—H23C	0.9600
			
N2—O3—C11	109.58 (10)	O4—C14—C15	103.25 (10)
C14—O4—C16	108.96 (10)	C13—C14—C15	114.24 (11)
C15—O5—C16	106.46 (10)	O4—C14—H14A	110.5
C8—N1—C11	114.83 (11)	C13—C14—H14A	110.5
C8—N1—C7	121.48 (11)	C15—C14—H14A	110.5
C11—N1—C7	122.64 (11)	O5—C15—C14	102.31 (11)
C13—N2—O3	109.12 (11)	O5—C15—H15A	111.3
C2—C1—C6	120.46 (13)	C14—C15—H15A	111.3
C2—C1—H1A	119.8	O5—C15—H15B	111.3
C6—C1—H1A	119.8	C14—C15—H15B	111.3
C3—C2—C1	120.08 (14)	H15A—C15—H15B	109.2
C3—C2—H2A	120.0	O5—C16—O4	105.73 (10)
C1—C2—H2A	120.0	O5—C16—C21	108.34 (11)
C2—C3—C4	120.08 (14)	O4—C16—C21	109.29 (11)
C2—C3—H3A	120.0	O5—C16—C17	111.57 (11)
C4—C3—H3A	120.0	O4—C16—C17	109.54 (11)
C3—C4—C5	119.91 (14)	C21—C16—C17	112.15 (11)
C3—C4—H4A	120.0	C16—C17—C18	111.64 (11)
C5—C4—H4A	120.0	C16—C17—H17A	109.3
C4—C5—C6	120.68 (13)	C18—C17—H17A	109.3
C4—C5—H5A	119.7	C16—C17—H17B	109.3
C6—C5—H5A	119.7	C18—C17—H17B	109.3
C5—C6—C1	118.79 (13)	H17A—C17—H17B	108.0
C5—C6—C7	119.86 (12)	C17—C18—C19	112.02 (12)
C1—C6—C7	121.23 (12)	C17—C18—H18A	109.2
N1—C7—C6	115.81 (10)	C19—C18—H18A	109.2
N1—C7—H7A	108.3	C17—C18—H18B	109.2
C6—C7—H7A	108.3	C19—C18—H18B	109.2
N1—C7—H7B	108.3	H18A—C18—H18B	107.9
C6—C7—H7B	108.3	C20—C19—C18	110.78 (12)
H7A—C7—H7B	107.4	C20—C19—H19A	109.5
O1—C8—N1	124.21 (13)	C18—C19—H19A	109.5
O1—C8—C9	125.55 (12)	C20—C19—H19B	109.5
N1—C8—C9	110.18 (11)	C18—C19—H19B	109.5
C10—C9—C8	102.28 (11)	H19A—C19—H19B	108.1
C10—C9—C23	110.89 (12)	C21—C20—C19	110.98 (12)
C8—C9—C23	109.38 (11)	C21—C20—H20A	109.4
C10—C9—C22	111.19 (12)	C19—C20—H20A	109.4
C8—C9—C22	112.38 (11)	C21—C20—H20B	109.4
C23—C9—C22	110.46 (12)	C19—C20—H20B	109.4
O2—C10—C9	127.14 (14)	H20A—C20—H20B	108.0
O2—C10—C11	122.72 (13)	C16—C21—C20	111.10 (11)
C9—C10—C11	110.08 (11)	C16—C21—H21A	109.4
N1—C11—O3	110.40 (11)	C20—C21—H21A	109.4
N1—C11—C12	116.94 (11)	C16—C21—H21B	109.4
O3—C11—C12	104.65 (11)	C20—C21—H21B	109.4
N1—C11—C10	102.50 (11)	H21A—C21—H21B	108.0
O3—C11—C10	109.28 (11)	C9—C22—H22A	109.5
C12—C11—C10	113.06 (11)	C9—C22—H22B	109.5
C13—C12—C11	101.63 (11)	H22A—C22—H22B	109.5
C13—C12—H12A	111.4	C9—C22—H22C	109.5
C11—C12—H12A	111.4	H22A—C22—H22C	109.5
C13—C12—H12B	111.4	H22B—C22—H22C	109.5
C11—C12—H12B	111.4	C9—C23—H23A	109.5
H12A—C12—H12B	109.3	C9—C23—H23B	109.5
N2—C13—C12	114.45 (11)	H23A—C23—H23B	109.5
N2—C13—C14	121.45 (12)	C9—C23—H23C	109.5
C12—C13—C14	124.11 (11)	H23A—C23—H23C	109.5
O4—C14—C13	107.61 (11)	H23B—C23—H23C	109.5
			
C11—O3—N2—C13	−5.71 (15)	O2—C10—C11—N1	175.05 (16)
C6—C1—C2—C3	0.9 (2)	C9—C10—C11—N1	−2.28 (14)
C1—C2—C3—C4	0.0 (2)	O2—C10—C11—O3	−67.84 (19)
C2—C3—C4—C5	−0.8 (2)	C9—C10—C11—O3	114.84 (12)
C3—C4—C5—C6	0.7 (2)	O2—C10—C11—C12	48.3 (2)
C4—C5—C6—C1	0.26 (19)	C9—C10—C11—C12	−129.05 (12)
C4—C5—C6—C7	176.37 (12)	N1—C11—C12—C13	115.85 (13)
C2—C1—C6—C5	−1.07 (19)	O3—C11—C12—C13	−6.62 (13)
C2—C1—C6—C7	−177.13 (13)	C10—C11—C12—C13	−125.45 (12)
C8—N1—C7—C6	81.76 (15)	O3—N2—C13—C12	1.00 (16)
C11—N1—C7—C6	−110.56 (14)	O3—N2—C13—C14	−179.24 (11)
C5—C6—C7—N1	106.45 (14)	C11—C12—C13—N2	3.71 (16)
C1—C6—C7—N1	−77.53 (16)	C11—C12—C13—C14	−176.04 (12)
C11—N1—C8—O1	−175.16 (13)	C16—O4—C14—C13	−136.03 (11)
C7—N1—C8—O1	−6.6 (2)	C16—O4—C14—C15	−14.88 (13)
C11—N1—C8—C9	2.36 (15)	N2—C13—C14—O4	−125.12 (14)
C7—N1—C8—C9	170.94 (11)	C12—C13—C14—O4	54.62 (17)
O1—C8—C9—C10	173.96 (13)	N2—C13—C14—C15	120.89 (14)
N1—C8—C9—C10	−3.52 (14)	C12—C13—C14—C15	−59.38 (17)
O1—C8—C9—C23	56.35 (18)	C16—O5—C15—C14	−36.76 (12)
N1—C8—C9—C23	−121.13 (13)	O4—C14—C15—O5	31.35 (13)
O1—C8—C9—C22	−66.72 (18)	C13—C14—C15—O5	147.89 (11)
N1—C8—C9—C22	115.80 (13)	C15—O5—C16—O4	28.29 (13)
C8—C9—C10—O2	−173.71 (16)	C15—O5—C16—C21	145.36 (11)
C23—C9—C10—O2	−57.2 (2)	C15—O5—C16—C17	−90.72 (12)
C22—C9—C10—O2	66.1 (2)	C14—O4—C16—O5	−7.12 (14)
C8—C9—C10—C11	3.47 (14)	C14—O4—C16—C21	−123.54 (12)
C23—C9—C10—C11	120.00 (12)	C14—O4—C16—C17	113.23 (12)
C22—C9—C10—C11	−116.68 (13)	O5—C16—C17—C18	−68.70 (14)
C8—N1—C11—O3	−116.37 (12)	O4—C16—C17—C18	174.58 (11)
C7—N1—C11—O3	75.20 (15)	C21—C16—C17—C18	53.04 (16)
C8—N1—C11—C12	124.19 (13)	C16—C17—C18—C19	−52.92 (16)
C7—N1—C11—C12	−44.24 (17)	C17—C18—C19—C20	54.69 (17)
C8—N1—C11—C10	−0.06 (14)	C18—C19—C20—C21	−56.37 (17)
C7—N1—C11—C10	−168.49 (11)	O5—C16—C21—C20	68.58 (14)
N2—O3—C11—N1	−118.92 (11)	O4—C16—C21—C20	−176.67 (11)
N2—O3—C11—C12	7.71 (14)	C17—C16—C21—C20	−54.99 (16)
N2—O3—C11—C10	129.07 (11)	C19—C20—C21—C16	56.61 (17)

### Hydrogen-bond geometry (Å, °)

**Table:**

*D*—H···*A*	*D*—H	H···*A*	*D*···*A*	*D*—H···*A*
C1—H1A···O1	0.93	2.48	3.1440 (18)	129
C14—H14A···O1^i^	0.98	2.50	3.4692 (17)	171
C15—H15A···Cg^ii^	0.97	2.89	3.6484 (15)	136

Symmetry codes: (i) −*x*, *y*+1/2, −*z*+1/2; (ii) *x*+1, *y*, *z*.
